# Association of soluble urokinase plasminogen activator receptor and epidermal growth factor with histopathological findings of kidney biopsy: a single-center study

**DOI:** 10.1186/s12882-025-04351-5

**Published:** 2025-07-25

**Authors:** Eman Nagy, Ahmed Almenshawy, Mostafa Abdelsalam, Ahmed M. Abd Elwahab, Ola M. Gharbia, Fatma El-Husseiny Moustafa, Nagy Sayed Ahmed, Nora A. Khreba

**Affiliations:** 1 Mansoura Nephrology and Dialysis Unit (MNDU), Internal Medicine Department, Faculty of Medicine, Mansoura University, Mansoura, Egypt; 2 Clinical Pathology Department, Faculty of Medicine, Mansoura University, Mansoura, Egypt; 3 Rheumatology and Rehabilitation Department, Faculty of Medicine, Mansoura University, Mansoura, Egypt; 4 Pathology Department, Faculty of Medicine, Mansoura University, Mansoura, Egypt

**Keywords:** Kidney, Fibrosis, Epidermal growth factor, EGF, Soluble urokinase plasminogen activator receptor, SuPAR, Histopathology, Biopsy

## Abstract

**Background:**

Despite the crucial role of kidney biopsy in the management of various kidney diseases, it has inherent limitations. Therefore, the search for non-invasive biomarkers as alternative diagnostic and prognostic tools is warranted. The aim of this study was to assess the association between soluble urokinase plasminogen activator receptor (suPAR) and epidermal growth factor (EGF) levels and various histopathological findings in patients undergoing kidney biopsy.

**Methods:**

This cross-sectional study involved patients who underwent kidney biopsies over a period of nine months. On the day of the biopsy, sociodemographic, clinical, and routine laboratory data were collected from patients’ medical records. Urine samples were obtained for measurement of urinary suPAR, EGF, and creatinine levels. Kidney biopsies were reviewed and interpreted by an expert nephropathologist.

**Results:**

A total of 82 patients (36 males) with a mean age of 36 years were included. The most common histopathological diagnosis was lupus nephritis (30.5%), followed by end-stage kidney disease (12%). Glomerulosclerosis (GS), tubular atrophy (TA), and interstitial fibrosis (IF) were present in 66%, 62%, and 74% of patients, respectively. Additionally, tubular injury, detached podocytes, and vascular fibrointimal thickening were observed in 30%, 5%, and 22% of patients, respectively. Both suPAR and EGF levels showed no statistically significant differences among varying degrees of GS, TA, and IF. However, urinary suPAR/creatinine was significantly higher in patients with tubular injury than in those without (*p* = 0.003). Its cut-off value to predict tubular injury was 0.08 with moderate sensitivity and specificity. Urinary EGF/creatinine was significantly lower in patients with detached podocytes than in those without (*p* = 0.028), whereas it was significantly higher in patients with vascular fibrointimal thickening than in those without (*p* = 0.043). Its cut-off value to predict vascular fibrointimal thickening was 0.88 with low-to-moderate sensitivity and moderate specificity.

**Conclusions:**

Both urinary suPAR/creatinine and urinary EGF/creatinine ratios were not associated with either glomerulosclerosis or IF/TA, and therefore, cannot substitute for kidney biopsy in the assessment of kidney fibrosis. Higher urinary suPAR was associated with tubular injury, suggesting its potential link with acute tubular damage. In contrast, lower urinary EGF levels were found to be associated with podocyte detachment. Additionally, increased urinary EGF was associated with vascular fibrointimal thickening, suggesting a possible role in vascular remodeling. These findings highlight associations that warrant further investigation in longitudinal studies.

## Introduction

Kidney biopsy is a crucial diagnostic tool for various kidney diseases, aiding in prognosis, assessment and therapeutic decision-making. However, it is an invasive procedure with potential complications [1]. These complications include hematuria, Page kidney, and subcapsular or retroperitoneal hematoma. In certain cases, these complications may require blood transfusion, radiological intervention, or surgical management [2]. Moreover, while kidney biopsy is essential for diagnosis in some cases, it may be contraindicated in conditions such as non-correctable coagulopathy, uncontrolled hypertension, kidney malformations, and thrombocytopenia [3]. This highlights the need for reliable biomarkers to facilitate noninvasive diagnosis of various kidney diseases.

It would be interesting to find some urinary or blood biomarkers that might be associated with certain kidney histopathological lesions that are evident mainly by kidney biopsy, e.g. tubulointerstitial injury and fibrosis, and podocyte injury. Kidney fibrosis is known to contribute to the progression of chronic kidney disease (CKD). Scarring can affect various compartments of the kidney structure, either individually or in combination, resulting in glomerulosclerosis (GS), tubular atrophy (TA), interstitial fibrosis (IF), and atherosclerosis [4]. Podocytopathy, a histopathological diagnosis indicating podocyte injury and encompassing a heterogeneous group of disorders [4], can occur in cases of glomerulopathies. Its prevalence is increasing, and it is expected to become one of the most significant causes of end-stage kidney disease (ESKD) [5].

The soluble urokinase-type plasminogen activator receptor (suPAR) is derived from the urokinase-type plasminogen activator receptor (uPAR), a membrane bound receptor expressed at the surface of multiple cell types such as immune cells and vascular endothelial cells. It can be detected in various body fluids including plasma, serum and urine [6]. Elevated plasma suPAR levels are associated with proteinuric kidney diseases, potentially due to β3 integrin activation in podocytes. This activation may lead to podocyte foot process effacement and subsequent disruption of the glomerular basement membrane [7]. Moreover, elevated levels of suPAR are associated with increased degrees of IF and TA [8–10].

On the other hand, epidermal growth factor (EGF) is a peptide growth factor from ascending limb of loop of Henle and the distal convoluted tubule. It might have a role in protecting kidney against fibrosis including IF and TA [11]. Reduced kidney function and CKD have been associated with lower baseline levels of urinary EGF [12]. This raises the question of whether EGF production in the kidney helps maintain kidney function and protects against fibrosis and inflammation, and whether a decline in EGF increases the risk of IF and TA [12].

The combined use of two biomarkers with opposing actions, such as EGF and suPAR, may provide additional insights compared to utilization of each of them alone. This study aimed to investigate the association of urinary suPAR and EGF with the histopathological findings in kidney biopsies of patients with various forms and degrees of kidney diseases.

### Patients and methods

The study design is cross-sectional that included all adult patients who underwent kidney biopsy at Mansoura University Hospital, Mansoura University, from June 2023 to February 2024. Kidney transplant recipients and patients with malignancies were excluded. A sample size of 81 participants were selected to achieve 90.1% power to reject the null hypothesis, assuming a population effect size of 0.35 and a significance level (alpha) of 0.05. Informed consent was obtained from all participants, and the study protocol was conducted in accordance with the Declaration of Helsinki and approved by the Ethics Committee at Mansoura Faculty of Medicine (Approval number R.24.03.2551).

Sociodemographic and clinical data, including age, sex, and history of diabetes mellitus (DM), hypertension, and systemic lupus erythematosus (SLE), were collected. Additionally, routine laboratory data at the time of kidney biopsy were obtained from patients’ medical records. These included complete blood count, liver enzymes, international normalized ratio (INR), erythrocyte sedimentation rate (ESR), serum creatinine, albumin, uric acid, calcium, and phosphorus, and quantitative urinary protein assessment.

### Urine sampling and biomarkers measurement

Urine samples were randomly collected by midstream clean catch sampling method at the day of kidney biopsy. Each urinary sample was gently shaken, and 3 mL was transferred into 2 plain tubes and centrifuged. The supernatant of the first tube was stored in − 80 freezing device till the biomarkers enzyme-linked immunosorbent assay (ELISA) assay. The other tube was used to measure urinary creatinine after sample dilution (1:50) and multiplying the result by dilution factor.

Urinary creatinine was measured using CXL PRO MISPA AGAPPE automated chemistry device. Urinary suPAR and urinary EGF were measured using ELISA techniques (DEVELOP Catalogue No.DLR-uPAR-Hu) and (DEVELOP Catalogue No. DLR-EGF-Hu) respectively. The kits utilized a double-antibody sandwich ELISA technique to assay the level of the two biomarkers in the samples.

### Kidney biopsy

**Fig. 1:**
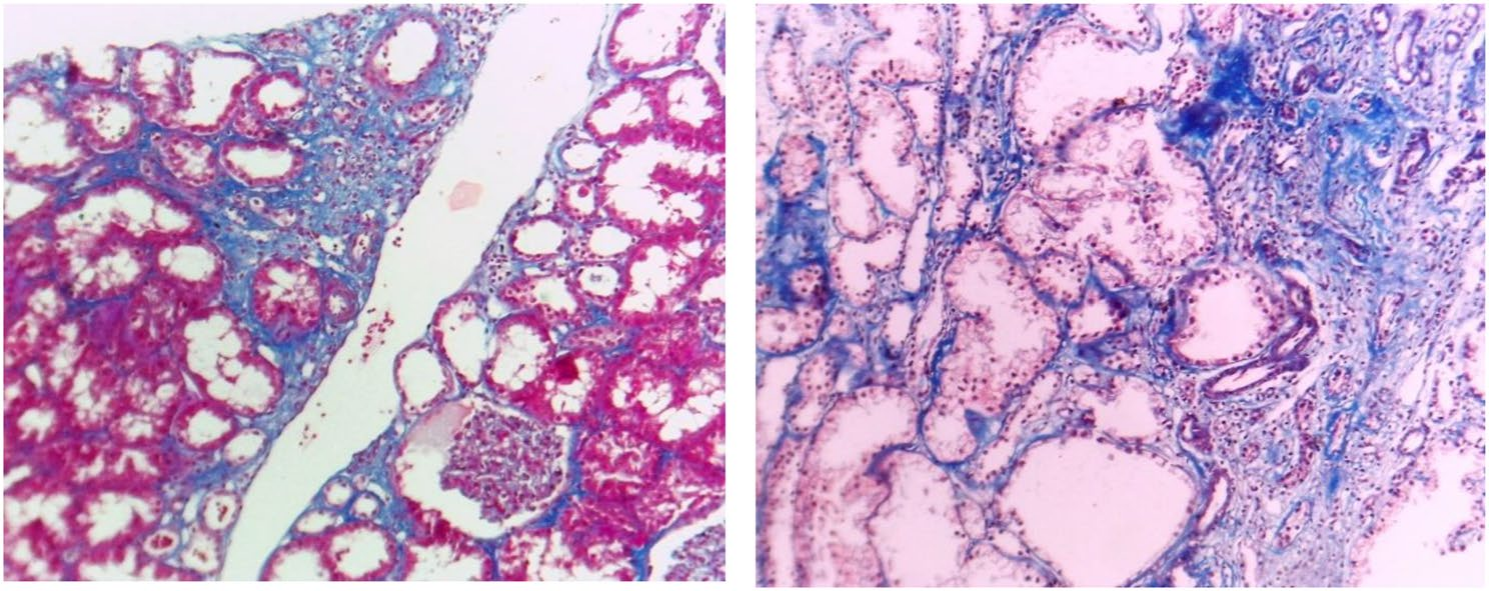
Interstitial fibrosis and tubular atrophy of less than 30%. (Masson trichrome stain x100)

**Fig. 2:**
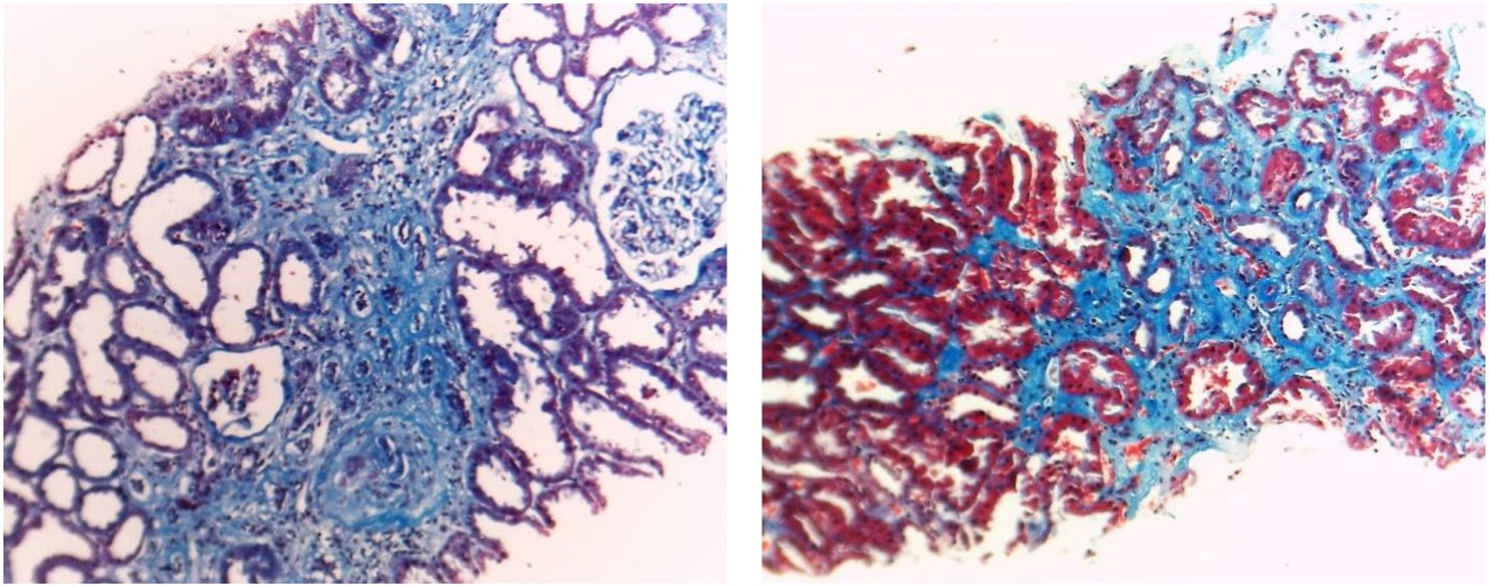
Interstitial fibrosis and tubular atrophy of about 30%. (Masson trichrome stain x100)

**Fig. 3:**
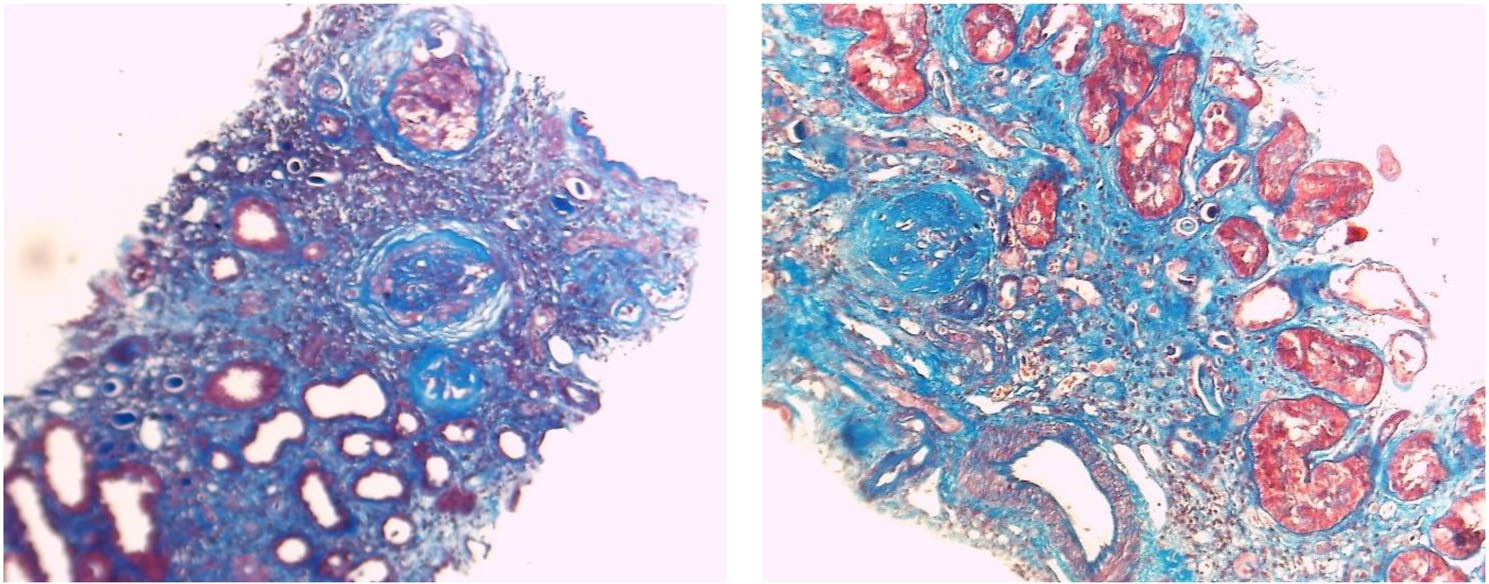
Severe fibrosis (> 50% of interstitium is fibrosed). Severe glomerulosclerosis (> 50% of glomeruli are totally sclerotic) (Masson trichrome stain x100)

Kidney biopsy samples were fixed in 10% neutral buffered formalin fixative, and 2 μm sections were prepared for light microscopy using hematoxylin and eosin, periodic acid-Schiff, Masson’s trichrome, and periodic acid silver methenamine staining. Histopathological evaluation included the severity of IF and TA, as well as the presence of detached podocytes (confirmed by electron microscopy). Mild GS, IF, and TA (Fig. 1) were defined as fibrosis involving less than 30%; moderate (Fig. 2) when fibrosis was between 30% and 50%; and severe (Fig. 3) when it exceeded 50% of tissue. The pathological interpretation was conducted by an expert nephropathologist blinded to the laboratory and biomarker data.

### Statistical analysis

Data were analyzed using SPSS (Statistical Package for the Social Sciences) version 29. Qualitative data were presented as numbers and percentages, while quantitative data were tested for normality using the Shapiro-Wilk test. The data were described as mean and standard deviation (SD) for normally distributed variables, and median and interquartile range (IQR) for non-normally distributed ones. Independent samples t-test and One Way Analysis of Variance (ANOVA) were used to compare parametric data for two and three groups, respectively. For non-parametric data, the Mann-Whitney and Kruskal-Wallis tests were applied for comparisons between two and three groups, respectively. Post-hoc analysis, such as the Tukey and Games-Howel tests, was performed following the Kruskal-Wallis and One Way ANOVA tests if significant differences were found. The Chi-square test was used to compare the differential distribution of categorical data. Binary logistic regression analysis was performed to identify independent predictors of tubular injury and vascular fibrointimal thickening. Receiver operating characteristic (ROC) curve was performed to allocate a cut-off point of suPAR/creatinine and EGF/creatinine ratios to predict tubular injury and vascular fibrointimal thickening and the cut-off point was chosen relying on the best possible specificity without sacrificing the sensitivity of choice. A *p*-value of less than 0.05 was considered statistically significant.

## Results

The study included 82 patients who underwent kidney biopsies at Mansoura University Hospitals. The median age of the participants was 36.5 years, and slightly more than half of them (56.1%) were females. Fifteen patients had diabetes mellitus, while 33 had hypertension. The median serum creatinine level was 2.5 mg/dL, and most patients were anemic, with a median hemoglobin level of 10.2 g/dL. The median quantitative proteinuria was more than 3 g/day, and the mean serum albumin level was below 3 g/dL. Table 1 provides detailed sociodemographic, medical, and laboratory data.

**Table 1:** Sociodemographic, clinical, and laboratory characteristics of the patients studied

	All patients (*n* = 82)
Age, years	36.5(25.8–49.3)
Sex:	
Male	36(43.9%)
Female	46(56.1%)
Smokers	24(29.3%)
DM	15(18.3%)
Duration of DM, years	8(3.8–11)
Hypertension	33(40.2%)
Duration of hypertension, years	2(0.1-5)
SLE	26(31.7%)
Serum creatinine, mg/dL	2.5(1.15–5.50)
WBCs, 10^9^/L	7.99(6.17–11.55)
Neutrophil, 10^9^/L	6.01(4.17–10.13)
Lymphocytes, 10^9^/L	1.58(0.99–2.25)
Neutrophil/lymphocytes ratio	3.69(2.16–7.75)
Blood hemoglobin, gm/dL	10.2(8.6–11.9)
Platelets, 10^9^/L	235(180–322)
Serum albumin, gm/dL	2.91 ± 0.88
Quantitative proteinuria, mg/day	3325(1625–5950)
ALT, U/L	22(20–31)
AST, U/L	22.5(21–35)
INR	1(1-1.09)
Serum uric acid, mg/dL	7.83 ± 2.82
Serum calcium, mg/dL	8.63 ± 0.77
Serum phosphorus, mg/dL	5.41 ± 1.68
ESR, mm/hour	83.74 ± 36.16

The data were expressed as N (%), mean ± SD, or median (IQR), as suitable

Abbreviations: ALT, Alanine Aminotransferase; AST, Aspartate aminotransferase; DM, Diabetes Mellitus; ESR, Erythrocyte Sedimentation Rate; INR, International Normalized Ratio; SLE, Systemic Lupus Erythematosus; WBCs, White Blood Cells

Among the biopsies studied, 30.5% were diagnosed as lupus nephritis, with various classes identified through kidney biopsy. Findings indicative of end-stage kidney disease (ESKD) was observed in 12% of the biopsies. ESKD is diagnosed when extensive fibrosis and sclerosis obscure the underlying cause of the original kidney disease. Additionally, more than 65% of the biopsies showed glomerulosclerosis, categorized as mild, moderate, and severe (21.7, 16.9, and 26.5% of them, respectively). Tubular atrophy was present in 51 patients, most of them were severe, interstitial fibrosis was similarly identified in 61 patients, most of them were also severe. Table 2 shows details of the kidney biopsy results.

**Table 2:** Kidney biopsy results

Number of glomeruli	17(12–25)
Percentage of sclerosed glomeruli	22(0–51)
Thickened glomerular capillary walls	15(18.3%)
Mesangial thickening	11(13.4%)
Endocapillary hypercellularity	15(18.3%)
Spikes or vacuolization	7(8.5%)
Hyaline lesion	14(17.1%)
Neutrophil/karyorrhexis	11(13.4%)
Fibrinoid necrosis	2(2.4%)
Detached podocytes	4(4.9%)
Cellular crescent	8(9.8%)
Fibrocellular crescent	3(3.7%)
Fibrous crescent	10(12.2%)
Interstitial infiltration	38(46.3%)
Interstitial fibrosis	61(74.4%)
Percentage of interstitial fibrosis	30(0–50)
Tubular injury	25(30.5%)
Tubular atrophy	51(62.2%)
Percentage of tubular atrophy	25(0–50)
Vascular fibrointimal thickening	18(22%)
Vascular necrosis	1(1.2%)
Glomerulosclerosis:	
Mild	18(21.7%)
Moderate	14(16.9%)
Sever	22(26.5%)
Tubular atrophy: Mild	13(15.7%)
Moderate	14(16.9%)
Sever	24(28.9%)
Interstitial fibrosis:	
Mild	17(20.5%)
Moderate	17(20.5%)
Sever	27(32.5%)
Diagnosis:	
Lupus nephritis	25(30.5%)
End stage kidney disease	10(12.2%)
Focal segmental glomerulosclerosis	8(9.8%)
Diabetic nephropathy	7(8.5%)
Acute tubular injury	4(4.9%)
Minimal change disease	4(4.9%)
Membranous nephropathy	3(3.7%)
Renal amyloidosis	3(3.7%)
Chronic tubulointerstitial nephritis	3(3.7%)
Membranoproliferative glomerulonephritis	2(2.4%)
Infection-related glomerulonephritis	2(2.4%)
Others	11(13.4%)

The data were expressed as N (%) or median (IQR), as suitable

There were statistically significant differences between mild, moderate, and severe glomerulosclerosis (GS) in relation to the presence of SLE, serum creatinine, and serum albumin. Patients with SLE had significantly milder degrees of GS. Serum creatinine and albumin levels were significantly higher in patients with severe GS compared to those with mild GS (post-hoc *p* = 0.005 and 0.012, respectively). Conversely, no statistically significant differences were found between the three degrees of GS concerning the urinary EGF/urinary creatinine or urinary suPAR/urinary creatinine (Table 3).

**Table 3:** Comparison between mild, moderate, and sever glomerulosclerosis

	Mild glomerulosclerosis (*n* = 18)	Moderate glomerulosclerosis (*n* = 14)	Severe glomerulosclerosis (*n* = 22)	*P* value
Age, years	41(27.5–50)	37(26.5–57)	33(25–41)	0.910
Sex:				0.742
Male	7(38.9%)	7(50%)	11(50%)	
Female	11(61.1%)	7(50%)	11(50%)	
Smokers	5(27.8%)	4(28.6%)	9(40.9%)	0.618
DM	3(16.7%)	2(14.3%)	3(13.6%)	0.963
Hypertension	8(44.4%)	7(50%)	14(63.6%)	0.456
SLE	11(61.1%)^1^	7(50%)	3(13.6%)^1^	**0.006**
Serum creatinine, mg/dL	1.70(1.25–3.03)^1^	2.65(0.98–4.70)	3.99(2.25–7.70)^1^	**0.006**
WBCs, 10^9^/L	8.75(6.98–12.25)	5.83(4.01–11.50)	7.85(5.80-11.43)	0.100
Neutrophil, 10^9^/L	7.14(4.51–10.12)	3.89(2.15–9.45)	6.04(4.52–11.34)	0.106
Lymphocytes, 10^9^/L	1.50(1.19–2.57)	1.13(0.55–2.20)	1.82(0.72–2.34)	0.626
Neutrophil/lymphocytes ratio	4.16(2.30–7.80)	3.27(1.81–6.53)	3.31(2.07–8.40)	0.721
Blood hemoglobin, gm/dL	10.75(9.08–11.85)	9.90(8.38–12.68)	9.00(8.20-11.38)	0.240
Platelets, 10^9^/L	241(152–346)	208(174–278)	228(204–323)	0.400
Serum albumin, gm/dL	2.55 ± 0.54^1^	2.77 ± 1.07	3.39 ± 0.82^1^	**0.039**
Quantitative proteinuria, mg/day	3250(1743–6900)	3471(1813–5300)	3309(1875–4820)	0.904
ALT, U/L	22(20–43)	22(20–60)	24(13–27)	0.887
AST, U/L	22(20–47)	22(22–37)	21(18–30)	0.126
INR	1(1-1.05)	1.06(1-1.13)	1.02(1-1.11)	0.452
Serum uric acid, mg/dL	9.13 ± 3.39	7.80 ± 1.19	7.72 ± 1.88	0.407
Serum calcium, mg/dL	8.43 ± 0.64	8.76 ± 0.40	8.49 ± 0.92	0.606
Serum phosphorus, mg/dL	4.40 ± 1.01	4.00 ± 0.71	5.70 ± 1.31	0.081
ESR, mm/hour	85(60–114)	60(31–89)	97(60–128)	0.088
Urinary EGF/urinary creatinine	0.28(0.17–0.55)	0.56(0.26–0.80)	0.68(0.30–1.41)	0.083
Urinary suPAR/urinary creatinine	0.05(0.03–0.09)	0.05(0.02–0.17)	0.07(0.03–0.17)	0.598

The data were expressed as N (%), mean ± SD, or median (IQR), as suitable

Similar superscripted numbers in the same row denote significant differences between the groups by the post-hoc analysis

Abbreviations: ALT, Alanine Aminotransferase; AST, Aspartate aminotransferase; DM, Diabetes Mellitus; EGF, Epidermal Growth Factor; ESR, Erythrocyte Sedimentation Rate; INR, International Normalized Ratio; SLE, Systemic Lupus Erythematosus; suPAR, soluble urokinase-type plasminogen activator receptor; WBCs, White Blood Cells

Similarly, patients with severe IF had significantly higher serum creatinine in comparison to those with mild IF (*p* = 0.003). In addition, patients with severe IF had significantly higher serum albumin than those with mild and moderate IF (*p* = 0.010, 0.039, respectively). Moreover, patients with severe IF had significantly higher serum phosphorus compared to those with mild IF (*p* = 0.030). On the other hand, there were no statistically significant differences between the three groups of IF as regards urinary EGF/urinary creatinine or urinary suPAR/urinary creatinine (Table 4).

**Table 4:** Comparison between mild, moderate, and sever interstitial fibrosis

	Mild interstitial fibrosis (*n* = 17)	Moderate interstitial fibrosis (*n* = 17)	Sever interstitial fibrosis (*n* = 27)	*P* value
Age, years	41(27.5–50)	37(26.5–57)	33(25–41)	0.236
Sex:				0.761
Male	8(47.1%)	6(35.3%)	12(44.4%)	
Female	9(52.9%)	11(64.7%)	15(55.6%)	
Smokers	4(23.5%)	6(35.3%)	8(29.6%)	0.754
DM	4(23.5%)	4(23.5%)	5(18.5%)	0.674
Hypertension	6(35.3%)	8(47.1%)	15(55.6%)	0.423
SLE	8(47.1%)	6(35.3%)	6(22.2%)	0.225
Serum creatinine, mg/dL	1.50(0.95–4.30)^1^	2.85(1.64–7.03)	3.99(2.50–6.20)^1^	**0.004**
WBCs, 10^9^/L	7.50(7.09–8.75)	8.80(5.46-12.00)	7.90(5.26–12.10)	0.885
Neutrophil, 10^9^/L	5.00(4.36–6.25)	8.06(4.62–10.07)	6.49(3.53–11.28)	0.486
Lymphocytes, 10^9^/L	1.76(1.36–2.61)	1.72(1.21–2.63)	1.10(0.67–1.91)	0.090
Neutrophil/lymphocytes ratio	2.83(2.03–3.69)	4.72(1.73–7.31)	5.59(2.77–9.47)	0.155
Blood hemoglobin, gm/dL	11.60(8.80–12.70)	10.70(8.55–11.95)	9.00(8.30–11.30)	0.141
Platelets, 10^9^/L	225(184–348)	218(175–321)	230(170–303)	0.948
Serum albumin, gm/dL	2.62 ± 0.70^1^	2.76 ± 0.80^2^	3.52 ± 0.88^1,2^	**0.006**
Quantitative proteinuria, mg/day	3455(2075–5858)	5500(2350–10330)	2388(939–4104)	0.051
ALT, U/L	22(12–41)	35(22–54)	22(20–25)	0.158
AST, U/L	23(19–29)	36(25–49)^1^	22(20–28)^1^	**0.018**
INR	1.06(1-1.20)	1(1-1.1)	1.01(1-1.1)	0.233
Serum uric acid, mg/dL	10.69 ± 3.20	9.76 ± 2.67	7.47 ± 1.71	0.112
Serum calcium, mg/dL	8.63 ± 0.60	8.49 ± 0.34	8.64 ± 0.85	0.855
Serum phosphorus, mg/dL	7.13 ± 0.74^1^	5.15 ± 0.48	4.90 ± 1.50^1^	**0.023**
ESR, mm/hour	80(70–110)	65(50–130)	100(55–125)	0.781
Urinary EGF/urinary creatinine	0.42(0.19–0.86)	0.57(0.28–2.75)	0.59(0.36–1.36)	0.511
Urinary suPAR/ urinary creatinine	0.06(0.02–0.16)	0.12(0.05–0.37)	0.05(0.02–0.11)	0.107

The data were expressed as N (%), mean ± SD, or median (IQR), as suitable

Similar superscripted numbers in the same row denote significant differences between the groups by the post-hoc analysis

Abbreviations: ALT, Alanine Aminotransferase; AST, Aspartate aminotransferase; DM, Diabetes Mellitus; EGF, Epidermal Growth Factor; ESR, Erythrocyte Sedimentation Rate; INR, International Normalized Ratio; SLE, Systemic Lupus Erythematosus; suPAR, soluble urokinase-type plasminogen activator receptor; WBCs, White Blood Cells

Regarding tubular atrophy (TA), serum creatinine was notably higher in patients with severe TA compared to those with mild and moderate forms (*p* = 0.001 and 0.024, respectively). Additionally, serum albumin levels were significantly higher in patients with severe than in those with mild TA (*p* = 0.022). Conversely, no statistically significant differences were found among the three TA groups concerning the urinary EGF/urinary creatinine or urinary suPAR/urinary creatinine (Table 5).

**Table 5:** Comparison between mild, moderate, and sever tubular atrophy

	Mild tubular atrophy (*n* = 13)	Moderate tubular atrophy (*n* = 14)	Sever tubular atrophy (*n* = 24)	*P* value
Age, years	41(29.5–55.5)	36(25.8–48.3)	32.5(25-40.8)	0.249
Sex:				0.911
Male	5(38.5%)	6(42.9%)	11(45.8%)	
Female	8(61.5%)	8(57.1%)	13(54.2%)	
Smokers	3(23.1%)	6(42.9%)	7(29.2%)	0.842
DM	3(23.1%)	1(7.1%)	4(16.7%)	0.737
Hypertension	5(38.5%)	6(42.9%)	13(54.2%)	0.615
SLE	8(61.5%)	6(42.9%)	6(25%)	0.089
Serum creatinine, mg/dL	1.10(0.85–2.50)^1^	2.10(1.45–2.85)^2^	3.75(2.58–5.68)^1,2^	**< 0.001**
WBCs, 10^9^/L	7.50(7.01–10.40)	9.00(6.35–11.80)	8.10(5.37–13.15)	0.995
Neutrophil, 10^9^/L	4.77(4.22–7.30)	8.06(5.42–10.07)	7.53(3.60-11.37)	0.536
Lymphocytes, 10^9^/L	2.23(1.42–2.79)^1^	1.72(1.30–2.73)	1.08(0.63–1.90)^1^	**0.035**
Neutrophil/lymphocytes ratio	2.33(1.87–3.16)	3.17(2.26–6.48)	6.92(3.14–9.73)	0.055
Blood hemoglobin, gm/dL	11.80(9.15–12.80)	11.25(8.48–12.15)	9.00(8.30-11.18)	0.111
Platelets, 10^9^/L	240(199–334)	248(198–323)	217(159–303)	0.691
Serum albumin, gm/dL	2.42 ± 0.64^1^	3.28 ± 0.90	3.45 ± 0.95^1^	**0.024**
Quantitative proteinuria, mg/day	3050(1948–5020)	4800(2843–6580)	2400(960–4500)	0.081
ALT, U/L	20(11–22)	22(20–43)	21.5(19.825)	0.355
AST, U/L	21.5(19.3–29.3)	36(22–47)	22(20–24)	0.179
INR	1.03(1-1.20)	1(1-1.04)	1(1-1.1)	0.145
Serum uric acid, mg/dL	5.8 ± 1.9	8.9 ± 2.6	7.7 ± 1.8	0.089
Serum calcium, mg/dL	8.45 ± 0.64	8.50 ± 0.13	8.62 ± 0.90	0.879
Serum phosphorus, mg/dL	8.00 ± 1.15	4.92 ± 1.38	4.92 ± 1.38	**0.012**
ESR, mm/hour	70(16.8–99.5)	76(46.3-122.5)	95(55–123)	0.497
Urinary EGF/urinary creatinine	0.42(0.15–0.82)	0.39(0.23–0.80)	0.59(0.37–1.31)	0.504
Urinary suPAR/urinary creatinine	0.07(0.02–0.50)	0.09(0.04–0.20)	0.05(0.02–0.10)	0.345

The data were expressed as N (%), mean ± SD, or median (IQR), as suitable

Similar superscripted numbers in the same row denote significant differences between the groups by the post-hoc analysis

Abbreviations: ALT, Alanine Aminotransferase; AST, Aspartate aminotransferase; DM, Diabetes Mellitus; EGF, Epidermal Growth Factor; ESR, Erythrocyte Sedimentation Rate; INR, International Normalized Ratio; SLE, Systemic Lupus Erythematosus; suPAR, soluble urokinase-type plasminogen activator receptor; WBCs, White Blood Cells

**Fig. 4:**
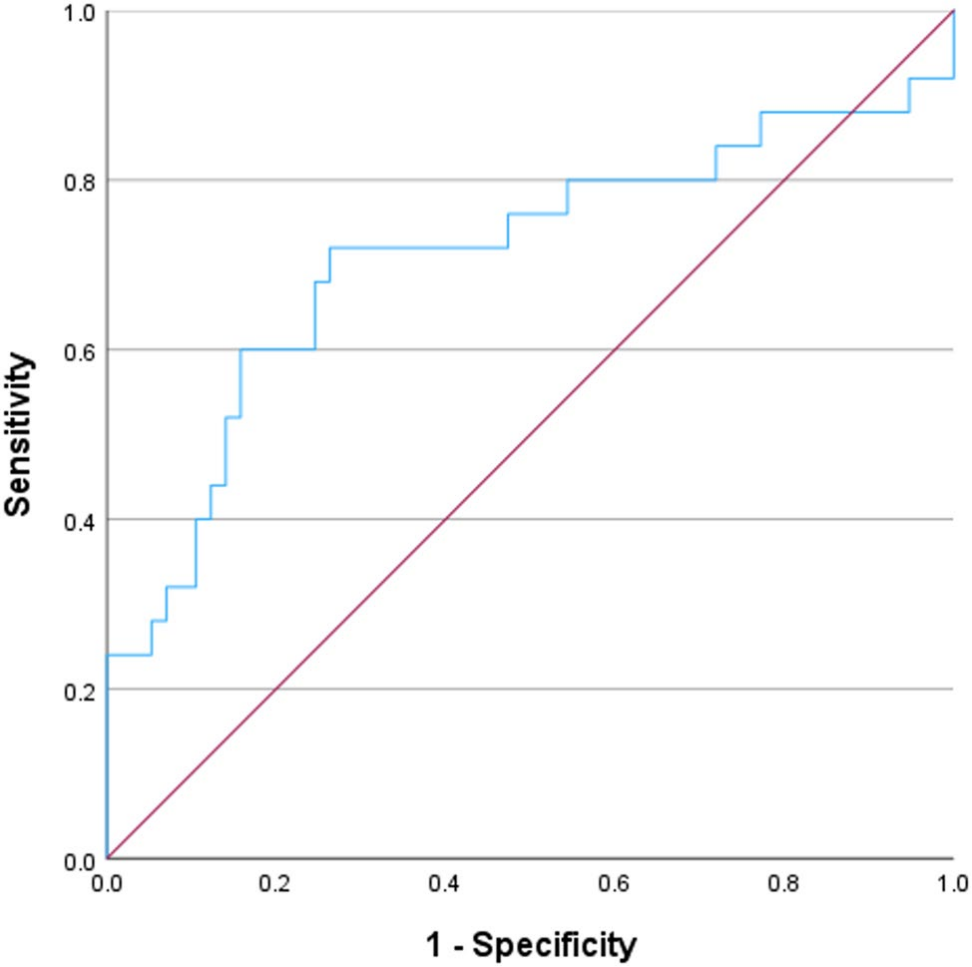
ROC curve for urinary suPAR/creatinine ratio as a predictor for tubular injury

Urinary suPAR/urinary creatinine was significantly higher in patients with, than in those without, tubular injury (median was 0.15 vs. 0.05, *p* = 0.003). Binary multivariate logistic regression was performed, including all variables found to be significant in univariate analysis, to identify independent predictors of tubular injury. The analysis showed that only serum creatinine remained a significant predictor (Table 6). By constructing ROC analysis to test urinary suPAR/urinary creatinine for prediction of tubular injury, it showed that area under curve (AUC) for prediction of tubular injury using urinary suPAR/urinary creatinine was 0.709 (*p* = 0.003). Using urinary suPAR/urinary creatinine optimal cut-off value of 0.08, as determined by the Youden index, the corresponding sensitivity and specificity for prediction of tubular injury were 72% and 74% respectively (Fig. 4).

**Table 6:** Binary multivariate logistic regression for predictors of tubular injury

	B	*p* value	OR	95% C.I.	
				Lower	Upper
Age	0.022	0.306	1.022	0.980	1.067
DM	-0.592	0.434	0.553	0.126	2.438
SLE	0.949	0.279	2.583	0.464	14.372
Serum creatinine	0.204	**0.027**	1.227	1.024	1.470
Urinary suPAR/creatinine ratio	0.836	0.082	2.307	0.900	5.910

C.I, confidence Interval; DM, Diabetes Mellitus; OR, Odds Ratio; SLE, Systemic Lupus Erythematosus; suPAR, soluble urokinase-type plasminogen activator receptor

**Fig. 5:**
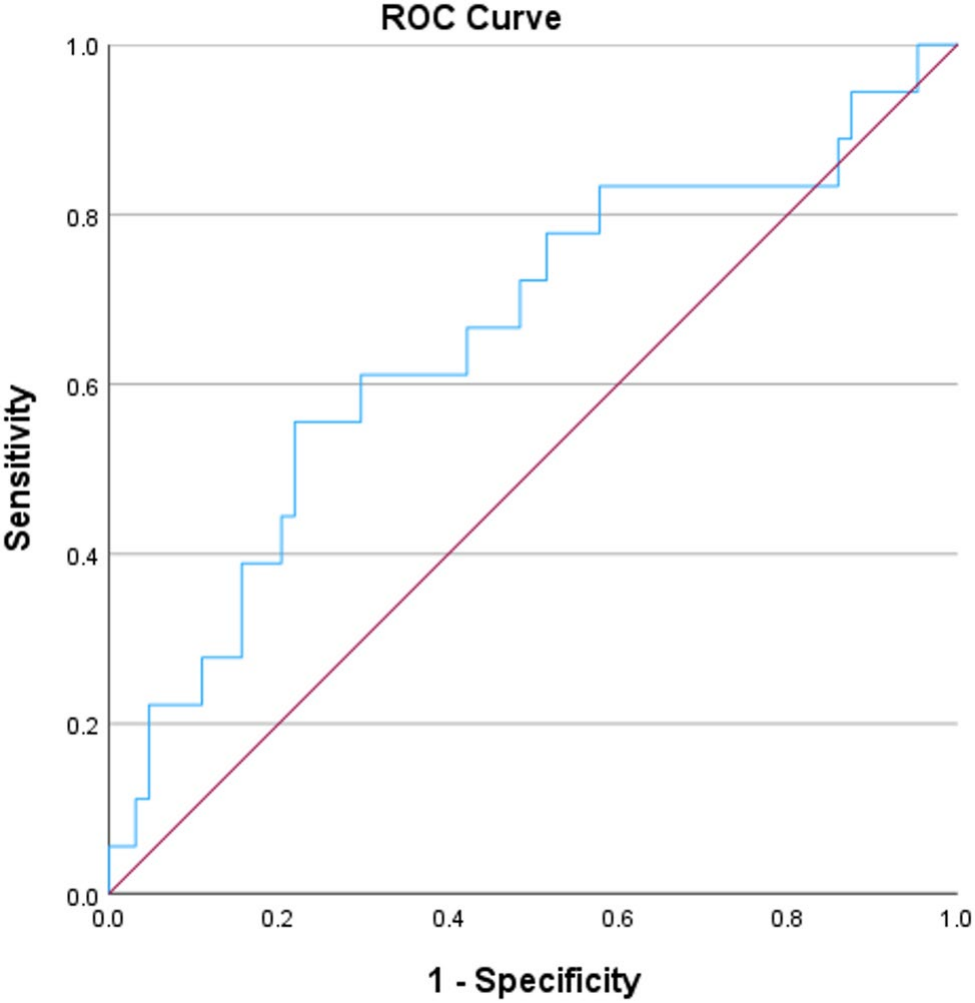
ROC curve for urinary EGF/creatinine ratio as a predictor for vascular fibrointimal thickening

On the other hand, urinary EGF/urinary creatinine was significantly lower in patients with, than in those without, detached podocytes (median was 0.14 vs. 0.54, *p* = 0.028). Conversely, it was significantly higher in patients with vascular fibrointimal thickening than in those without this lesion (*p* = 0.043). Binary multivariate logistic regression was performed, including all variables found to be significant in univariate analysis, to identify independent predictors of vascular fibrointimal thickening. The analysis excluded all the variables from being significant predictors of vascular fibrointimal thickening (Table 7). The ROC curve was performed to assess the ability of urinary EGF/urinary creatinine to predict vascular fibrointimal thickening and resulted in AUC of 0.657 (*p* = 0.043). Using urinary suPAR/urinary creatinine optimal cut-off value of 0.88, as determined by the Youden index, the corresponding sensitivity and specificity for prediction of tubular injury were 56% and 78% respectively (Fig. 5).

**Table 7:** Binary multivariate logistic regression for predictors of vascular fibrointimal thickening

	B	*p* value	OR	95% C.I.	
				Lower	Upper
Age	0.041	0.072	1.042	0.996	1.089
DM	-0.990	0.197	0.372	0.083	1.671
Serum creatinine	0.200	0.020	1.221	1.032	1.445
Urinary EGF/creatinine ratio	0.095	0.554	1.100	0.803	1.506

C.I, confidence Interval; DM, Diabetes Mellitus; OR, Odds Ratio; EGF, Epidermal Growth Factor

## Discussion

This study included 82 patients who underwent kidney biopsies at Mansoura University Hospitals. Lupus nephritis was the most common histopathological diagnosis. Glomerulosclerosis was observed in 54 patients, tubular atrophy in 51 patients, and interstitial fibrosis in 71 patients. Additionally, 25 patients exhibited tubular injury, while detached podocytes were noted in 4 patients.

Our sample had a relatively younger median age (36.5 years), which reflects the demographics of patients having native kidney biopsy at our tertiary care institution, where a large proportion of cases involve autoimmune and primary glomerular disorders that manifest at a young age. Although age can influence both histopathological characteristics and biomarker levels, we found no significant link between age and urinary suPAR or EGF in our research. On the contrary, in a study by *Wei et al.*, suPAR levels were predominantly linked to podocyte injury in an elderly population [9]. Similarly, *Xu et al.* [13], *Postalcioglu et al.* [14], and *Ju et al.* [15] found age-related reductions in urinary EGF, typically in the presence of increasing CKD and tubulointerstitial fibrosis.

Soluble urokinase plasminogen activator receptor (suPAR) is the soluble form of the membrane-bound urokinase receptor. It is expressed by a variety of cells, including endothelial cells, podocytes, and activated immune cells [16]. In the present study, urinary suPAR/urinary creatinine was significantly higher in patients with tubular injury than those without. suPAR might afflict podocytes and tubular cells via various mechanisms including promoting kidney fibrosis in an integrin-dependent manner [17]. In addition, it may contribute to kidney damage through various mechanisms and has been identified as a marker of acute tubular injury [16, 17]. It may be due to increased suPAR release from injured tubular cells, impaired renal clearance, or associated tubulointerstitial inflammation.

In the current study, urinary suPAR/creatinine levels did not show statistically significant differences among the three degrees of glomerulosclerosis, tubular atrophy and interstitial fibrosis. This finding aligns with *Spinale et al.*, who found no correlation between serum or urinary suPAR concentrations and focal segmental glomerulosclerosis (FSGS) histopathology [18]. While *Cathelin et al.*. demonstrated that neither short-term nor prolonged administration of suPAR, nor its deposition on glomerular structures, was sufficient to induce proteinuria in mice [19]. Furthermore, their inducible transgenic mouse model, with a sustained two- to three-fold increase in circulating suPAR, did not develop proteinuria.

Conversely, *Wei et al.* provided evidence that increased recombinant suPAR concentrations can induce proteinuria, foot process effacement, and FSGS histopathology. These effects were observed in both short-term and prolonged exposure experiments using plasminogen activator, urokinase receptor (PLAUR)-null mice treated with recombinant suPAR [9]. It is possible that *Wei’s* findings of proteinuria and segmental sclerosis were due to chronic suPAR expression.

Our findings contradict some of the existing literature, where elevated suPAR levels have been primarily linked to podocyte injury and proteinuria [9], whereas urinary EGF is typically associated with tubular regeneration and anti-fibrotic responses [15]. One probable explanation is that suPAR has broader pathogenic consequences beyond podocytes, such as tubular inflammation and fibrosis which indicate the need for further evaluation of its role.

In the current study, urinary EGF/urinary creatinine was significantly lower in patients with detached podocytes than those without. Strategies that protect podocytes from harm before depletion are desirable to prevent the onset and progression of glomerular disorders, because podocytes cannot be replicated [20]. To the best of our knowledge, no established markers have been identified that are specifically upregulated in injured podocytes in human tissues. EGF is the typical epidermal growth factor receptor (EGFR) ligand, which is mainly originated from the kidney [21]. Studies revealed that EGF accelerate the renal repair after an injurious insult [22, 23] and EGFR deletion delays recovery of kidney injury [13, 24]. In addition, *Li et al.* reported that EGF alone could inhibit podocytes apoptosis and injury in high glucose environment. In addition, they reported that EGFR expression is reduced in renal tissue of rats with diabetic nephropathy and high glucose-induced podocytes [25]. On the other hand, *Rayego et al.* reported that EGFR activation accelerates renal damage in CKD by initiation of a fibrotic -related process [26]. Our results suggest that EGF may serve as a protective marker against podocyte injury in human glomerular diseases. Low urine EGF levels in the presence of podocyte injury could indicate a general deficit in renal regeneration signaling across different renal compartments or a broader disturbance in renal homeostasis. Variations in biopsy timing, disease stage, or local tissue expression versus urinary biomarker levels might also contribute to these discrepancies. However, the underlying mechanisms through which EGF prevents podocyte apoptosis and injury require further investigation.

In the current study, the urinary EGF/creatinine ratio was significantly higher in patients with vascular fibrointimal thickening compared to those without. This may be attributed to the role of EGF in the proliferation and migration of vascular smooth muscle cells. Consequently, elevated EGF levels could promote increased vascular remodeling and intimal hyperplasia [27, 28]. EGF can act as an inflammatory mediator, and its elevation in fibrointimal thickening reflects the attempt of the body to repair the vascular tissues [29]. In addition, it have been shown that EGF is present in atherosclerotic plaques and that it is a potent mitogenic and chemotactic factor for vascular smooth muscle cells [30, 31].

A decrease in urinary EGF has been shown to correlate with IF-TA [32] and may predispose individuals to progressive kidney disease [33]. Additionally, the administration of exogenous EGF has demonstrated a protective effect on kidney function in mouse models with experimentally induced kidney impairment [34, 35]. However, in this study, no statistically significant differences were observed in the urinary EGF/creatinine ratio among different degrees of glomerulosclerosis, tubular atrophy, and interstitial fibrosis. This may be attributed to variations in sampling methods, differences in study methodology, and the relatively small sample size.

This study evaluated two urinary biomarkers as potential noninvasive indicators for the histopathological diagnosis of patients undergoing kidney biopsy: suPAR as a marker of fibrosis and EGF as a marker of repair. A substantial number of kidney biopsies were analyzed, offering insights into the relationship between urinary biomarkers and various histopathological lesions. However, the study had several limitations, including a relatively small sample size, a cross-sectional design, and its conduction at a single center, which may limit generalizability. Additionally, potential confounders such as medication use, and disease severity may not have been fully accounted for.

To date, evidence regarding potential novel biomarkers to assess kidney fibrosis instead of kidney biopsy is still very scattered and none of the biomarkers are routinely employed in clinical practice. Thus, further studies are needed. Combining different biomarkers, rather than using a single one, could be the best direction to follow.

## Conclusion

Both urinary suPAR/creatinine and urinary EGF/creatinine ratios were not associated with either glomerulosclerosis or IF/TA, and therefore, cannot substitute for kidney biopsy in the assessment of kidney fibrosis. Higher urinary suPAR was associated with tubular injury, suggesting its potential link with acute tubular damage. In contrast, lower urinary EGF levels were found to be associated with podocyte detachment. Additionally, increased urinary EGF was associated with vascular fibrointimal thickening, suggesting a possible role in vascular remodeling. These findings highlight associations that warrant further investigation in longitudinal studies.

## Data Availability

The datasets used and/or analyzed during the current study are available from the corresponding author on reasonable request.
